# Cr/AlCrNbSiTiN/AlCrNbSiTiO Gradient Nano-Multilayer Coatings with Excellent Solar Absorption and Photothermal Conversion Properties

**DOI:** 10.3390/nano16120713

**Published:** 2026-06-10

**Authors:** Qingyu Wang, Sheng Liu, Shikun Liu, Yanxiong Xiang, Changwei Zou

**Affiliations:** 1 School of Physics Science and Technology, Lingnan Normal University, Zhanjiang 524048, China; qingyuwangg@163.com (Q.W.);; 2 Research Center for Marine Metal Surface Enhancement in Western Guangdong, Lingnan Normal University, Zhanjiang 524048, China

**Keywords:** magnetron sputtering, AlCrNbSiTi high-entropy alloy, photothermal conversion, thermal stability

## Abstract

High-entropy alloys exhibit a broad light-responsive spectrum, spanning the ultraviolet to visible range, and their light absorption coefficient is significantly higher than that of traditional binary oxides. Cr/AlCrNbSiTiN/AlCrNbSiTiO gradient nano-multilayer coatings with excellent solar selective absorption properties are prepared using ion source enhanced magnetron sputtering. The effects of thickness of the absorption layer of AlCrNbSiTiN (3/4/5 min, denoted as S-3/4/5) are systematically investigated. It is worth noting that nano-multilayer coatings of S-3, S-4, and S-5 exhibit nearly perfect absorption rates of 0.9847, 0.9888, and 0.9879, respectively. The TEM images shows clear interfaces between the various coating layers, exhibiting a gradient structure that combines nanocrystalline and amorphous characteristics. From the substrate to the surface, there is an increase in the content of nanocrystalline phases, coarsening of grain sizes, and a decrease in the amount of amorphous phases. The primary absorption layer of AlCrNbSiTiN displays a typical face-centered cubic nitride structure. The XPS analysis reveals that the high-valent oxides (Nb^5+^, Cr^6+^) ensure thermal stability, whereas mixed valence states of Cr^3+^/Cr^6+^ may enhance visible light absorption through multi-electron transitions. This study analyzes how both the thickness of absorbing layers and high-temperature annealing affect the optical properties and photothermal conversion performance of AlCrNbSiTiN-based high-entropy coatings, which provides valuable insights for developing high-performance selective absorbers.

## 1. Introduction

Since the commencement of the Industrial Revolution, the rapid evolution of technology has considerably elevated societal productivity. However, this development has been accompanied by increasingly grave energy consumption concerns. Currently, fossil fuels make up more than 80% of the global energy consumption framework [1], resulting in pressing matters such as ecological degradation and global climate change. Solar radiation serves as the Earth’s primary energy source, with roughly 4 × 10^15^ MW of solar energy reaching the planet’s surface each year-equivalent to 36 trillion tons of standard coal, or 2000 times the total annual global energy consumption [2,3]. Against this backdrop, solar energy, as a pollution-free, clean, and renewable energy source, has emerged as a critical choice for attaining the “dual carbon” goals. The core of solar energy photothermal conversion lies in the capture and conversion of solar radiation by selective absorption materials. However, solar energy is characterized by low energy density and high susceptibility to dissipation, which necessitates the development of high-performance absorption materials to enhance its utilization efficiency [4]. According to Planck’s law, the sun can be regarded as a black body radiation source at 5778 K, with its primary energy distributed in the 0.3–2.5 μm wavelength range [5]. An ideal selective absorption coating should achieve complete absorption (α≈1) in this range while maintaining high reflection (R≈1) in the 2.5–25 μm infrared range to minimize heat loss. Currently, common coating types include intrinsic absorption coatings, interference absorption coatings, and metal-dielectric composite coatings. Among these, bimetallic ceramic coatings that operate based on interference and ceramic-metal composite mechanisms have been widely applied due to their excellent performance [6,7].

High-entropy alloys (HEAs), as a novel type of multi-principal alloy material, have attracted significant attention since the pioneering research by Yeh et al. in 2004 [8,9]. Unlike conventional alloys, HEAs exist as solid solutions, and their multi-principal element characteristics result in atomic size mismatch, causing significant lattice distortion. This lattice distortion effect endows the material with superior properties [10,11,12,13]. By leveraging the “cocktail effect” to tailor alloy compositions, material performance can be further enhanced. For example, the addition of Si can improve the oxidation resistance of FeCoNiCrSi_x_ coatings [14]. Nitrogen (N) exerts a pronounced solid solution strengthening effect and can impede dislocation movement, allowing nitrogen-containing alloys to exhibit higher recrystallization and nucleation rates [15,16]. Interstitial atoms (N, C, O) are capable of refining grain size and enhancing strength [17]. Ming et al. fabricated two non-equivalent AlCrNbSiTi high-entropy nitride coatings via magnetron sputtering technology [18]. Both coatings featured a face-centered cubic (fcc) NaCl-type structure and demonstrated excellent plasticity and toughness, rendering them suitable for energy absorption devices. In addition, materials such as TiZrHfNbTaO_5_N_3_ exhibited a narrow bandgap of 1.6 eV, which was attributed to the synergistic effects of oxygen/nitrogen vacancy defects and 2p orbital hybridization, which significantly facilitated carrier generation [19]. We have also synthesized self-organized AlCrNbSiTiN/CrN nano-multilayer high-entropy nitride coatings using arc ion plating technology by regulating the spatial distribution of plasma components, which exhibited superior mechanical, frictional, and corrosion resistance [20].

Compared to conventional binary metal oxides and nitrides, these high-entropy materials not only demonstrate a broad-spectrum optical response range covering both the ultraviolet and visible light regions but also possess a notably higher optical absorption coefficient than traditional binary oxides such as TiO_2_, ZrO_2_, HfO_2_, Nb_2_O_5_, and Ta_2_O_5_ [21,22,23]. This renders them promising candidates for the development of high-performance selective absorption coating materials. This study centers on the application of high-entropy alloy coatings in the field of solar energy photothermal conversion, with the aim of providing innovative material solutions for advancing renewable energy technologies.

## 2. Experiment and Characterization

### 2.1. Experimental Materials and Methods

This experiment employed magnetron sputtering technology, using a KYVAC-200CK desktop coating system (Shenzhen Supu Instruments Co., Ltd., Shenzhen, China) to deposit high-entropy photothermal conversion coatings onto Si substrates. The Si substrates were N-type (111) single-crystal silicon wafers with dimensions of 2 cm × 2 cm × 0.05 cm. Prior to the experiment, the substrates were placed in Petri dishes and sequentially cleaned in an ultrasonic cleaner with deionized water and anhydrous ethanol, each for 10 min. After drying, the substrates were transferred into the vacuum chamber. The targets used for coating preparation included a Cr target (purity 99.5%, diameter 10.0 cm) and an AlCrNbSiTi target (purity 99.5%, atomic ratio Al:Cr:Nb:Si:Ti = 22:34:11:11:22). The Cr target was sputtered under Ar atmosphere, while the high-entropy target was sputtered under mixed gas atmospheres of Ar/N_2_ and Ar/O_2_ at a flow rate ratio of 4:1. All gases used in the experiment had a purity of over 99.999%. The deposition parameters for each layer are listed in Table 1. The photothermal coating samples were named Si-ACNSTN-3, Si-ACNSTN-4, and Si-ACNSTN-5 according to the deposition time of the main absorption layer.

### 2.2. Structural and Characterization of Coatings

Based on the service conditions of photothermal conversion coatings, annealing experiments on AlCrNbSiTiON-based nanogradient coatings were carried out in an SK-G06163-2T box-type muffle furnace (Shanghai Yiheng Scientific Instrument Co., Ltd., Shanghai, China) under an air atmosphere. The temperature was raised at a rate of 10 °C per minute to 500 °C, held constant for 60 min, and then allowed to cool naturally to room temperature. To investigate the phase composition of the samples before and after heat treatment, an X’Pert Pro X-ray diffractometer (Malvern Panalytical, Almelo, The Netherlands) was used. The surface and cross-sectional morphologies of the samples were examined using a Hitachi SU2800 field emission scanning electron microscope (FE-SEM, Hitachi High-Tech Co., Hitachinaka-shi, Ibaraki-ken, Japan). Prior to SEM imaging, the samples were sputtered with gold for 30 s to improve conductivity. The nanostructure and crystallinity of the coatings were observed using a Talos F200X transmission electron microscope (TEM, Thermo Scientific, Waltham, MA, USA) at an accelerating voltage of 200 kV. The chemical states and elemental composition of the coatings were analyzed using an AXIS SUPRA X-ray photoelectron spectrometer (XPS, Shimadzu Co., Kyoto, Japan) with monochromatic Al K_α_ radiation (1486.6 eV).

To evaluate the optical performance of the coatings, a self-developed composite temperature measurement system was employed to monitor the dynamic temperature rise process in real time under standard 1 sun (AM1.5G) illumination. The system comprises a high-stability xenon lamp source, a TASi TA612C K-type thermocouple thermometer(Suzhou Tians Electronic Industrial Co., Ltd., Suzhou, China), a cooled sample stage, and data acquisition software. During the test, the heating duration was set to 3000 s and the cooling duration to 1500 s. The absorption rates of the coatings were measured using a UV-3600Plus ultraviolet-visible-near-infrared spectrophotometer (Shimadzu Co., Kyoto, Japan). The test parameters were set as follows: wavelength range of 200–2500 nm, resolution of 0.1 nm, wavelength display unit of 0.01 nm, and scan speed of 1 nm/min. The measured reflectance spectra were mathematically processed using numerical integration to accurately calculate the absorption rate (α) of the coating material, as represented by the following formula [24]:

$$\alpha =\frac{\int_{0.3}^{2.5}I_{sol}\left(\lambda \right)[1-R(\lambda )]d\lambda }{\int_{0.3}^{2.5}I_{sol}\left(\lambda \right)d\lambda }$$

where λ is the wavelength, R(λ) is the measured reflectance, and $I_{sol}$ represents the solar radiation power.

## 3. Results and Discussion

### 3.1. Optical Performance of the Coatings

**Figure 1:**
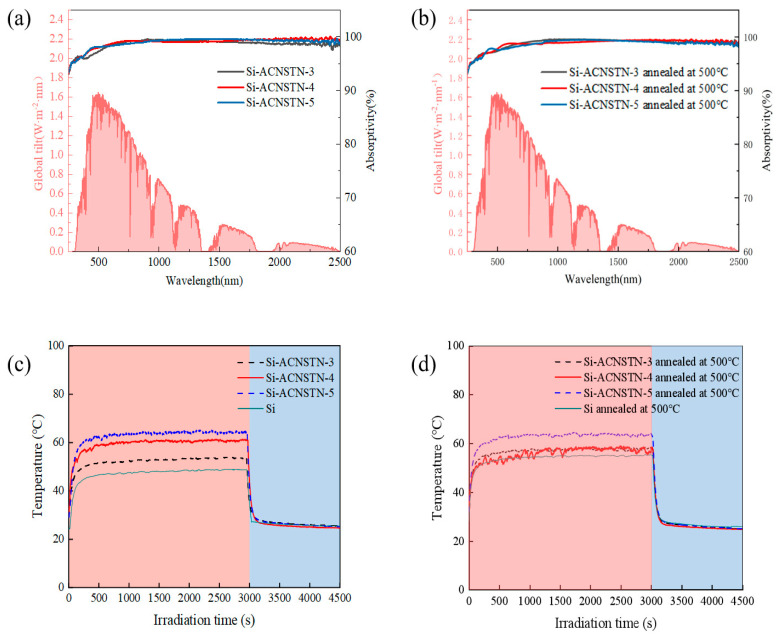
Reflectance spectra (**a**,**b**) and temperature-rise/temperature-fall curves (**c**,**d**) of the coating in the as-deposited state and after 1 h of heat treatment at 500 °C.

Figure 1 presents the reflectance spectra and temperature rise-fall curves of the coatings in both their as-deposited state and after undergoing 1 h heat treatment at 500 °C. In Figure 1a, the full-spectrum light absorption rates of samples Si-ACNSTN-3, Si-ACNSTN-4, and Si-ACNSTN-5 reach 0.9847, 0.9888, and 0.9879 respectively, showing outstanding spectral absorption performance-with Si-ACNSTN-4 delivering the optimal results. Figure 1b displays the absorption spectra of the samples post-annealing. The findings reveal that the light absorption performance of the samples did not experience a significant decline after annealing. Calculations show that the full-spectrum light absorption rates of the annealed samples Si-ACNSTN-3, Si-ACNSTN-4, and Si-ACNSTN-5 are 0.9868, 0.9864, and 0.9850 respectively. In comparison to their as-prepared state, the absorption rate of the Si-ACNSTN-3 multilayer coating increased by 0.21%, while those of Si-ACNSTN-4 and Si-ACNSTN-5 decreased by a mere 0.24% and 0.29% respectively. This demonstrates that the multilayer coating samples possess excellent thermal stability.

The amorphous structure of AlCrNbSiTi-based nitrides enables additional charge trapping at interface defects, thereby enhancing the oscillation of photo-generated carriers [25,26]. Moreover, the high-entropy alloy nitride material system possesses intrinsic metallic properties. As nitrogen content increases, the ionic bond character becomes stronger, accompanied by enhanced electron localization and increased covalent bonding. These factors contribute to the formation of unique electronic structures in high-entropy alloy nitrides, which facilitate free carrier absorption and surface plasmon resonance effects [27]. The intrinsic absorption of the high-entropy alloy nitride primary absorption layer synergizes with the interference cancellation generated by the low-metal-content secondary absorption layer. This integrated mechanism endows the Cr/AlCrNbSiTiN/AlCrNbSiTiO multilayer coating with excellent solar energy collection capabilities.

The surfaces of the Si-ACNSTN-3, Si-ACNSTN-4, and Si-ACNSTN-5 samples were rapidly heated from room temperature (28 °C) to stabilize at peak temperatures of 58.9 °C, 61.6 °C, and 65.1 °C, respectively. In contrast, the Si wafer exhibited a slower temperature rise, stabilizing at approximately 53.6 °C. When the xenon lamp was turned off, the surface temperatures of all samples dropped immediately and stabilized back at 28 °C. The as-deposited samples demonstrated a rapid thermal response within 500 s, with temperature increases of 25.9 °C, 29.4 °C, and 33.2 °C-all exceeding the 22.5 °C rise observed for the Si substrate. This suggests that the surface of the HEA gradient coating plays a key role in the efficient conversion of light energy into heat. For the heat-treated samples, the maximum temperature rise of the annealed Si-ACNSTN-3 sample increased from 53.8 °C (before annealing) to 58.7 °C, indicating a significant improvement in photothermal conversion efficiency. A possible explanation for this phenomenon is that during the annealing process, high temperatures promote the rearrangement of lattice atoms, reducing defects such as vacancies, dislocations, and impurity clusters. The reduction in defects can significantly extend the lifetime of photogenerated carriers, thereby enhancing their participation in the photothermal conversion process. For the annealed Si-ACNSTN-4 sample, the maximum temperature rise decreased from 61.6 °C before annealing to 58.9 °C, indicating that its photothermal conversion efficiency was not significantly affected by temperature. For the annealed Si-ACNSTN-5 sample, the maximum temperature rise was 64.9 °C, representing a minor change of only 0.2 °C compared to its pre-annealing value of 65.1 °C. Additionally, it exhibited a rapid photothermal response rate within the first 800 s, which far exceeded that of the annealed Si substrate. These results demonstrate that the solar-selective absorption coating samples prepared in this experiment have excellent thermal stability.

### 3.2. Morphologies of the Coatings

**Figure 2:**
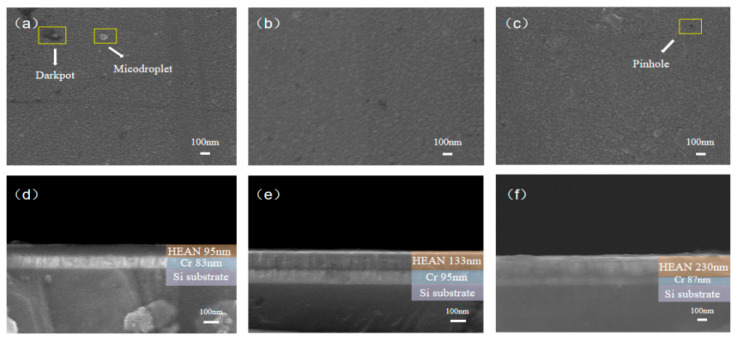
Surface (**a**–**c**) and cross-sectional (**d**–**f**) morphologies of the deposited coatings: Si-ACNSTN-3 (**a**,**d**), Si-ACNSTN-4 (**b**,**e**), and Si-ACNSTN-5 (**c**,**f**).

The morphologies of the as-deposited coatings are shown in Figure 2. These coatings exhibited common defects, such as sub-100 nm pinholes and irregular small particles-features typical of films deposited via magnetron sputtering, arising from ion bombardment and uneven magnetic field distribution. Overall, the surfaces were smooth, uniform, and dense, with no prominent large defects. Cross-sectional SEM images revealed distinct interfaces between the metallic Cr infrared reflective layer and the high-entropy alloy nitride coating. Cross-sectional images also showed slight changes at the interface between the Cr layer and the HEAN coating, where the interface became less distinct, indicating that annealing had a minimal impact on the microstructure.

**Figure 3:**
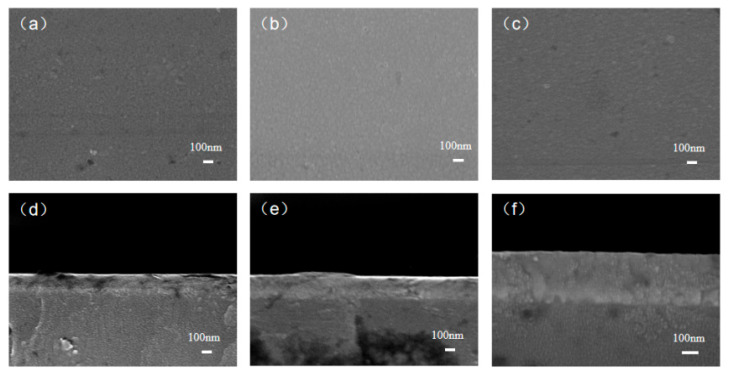
Surface (**a**–**c**) and cross-sectional (**d**–**f**) morphologies of the coatings on the samples after 1 h of heat treatment at 500 °C. Si-ACNSTN-3 (**a**,**d**), Si-ACNSTN-4 (**b**,**e**), and Si-ACNSTN-5 (**c**,**f**).

**Figure 4:**
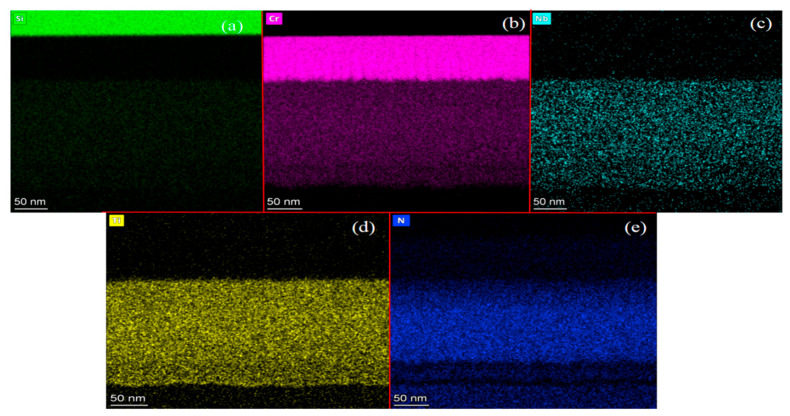
EDS mapping images of Si-ACNSTN-4: Si (**a**), Cr (**b**), Nb (**c**), Ti (**d**) and N (**e**).

Figure 3 displays the surface and cross-sectional microstructures of the Cr/AlCrNbSiTiN/AlCrNbSiTiO multilayer coating following heat treatment at 500 °C for 1 h in an air atmosphere. In comparison to the as-deposited samples, the annealed ones featured a uniform, smooth surface free of cracks or new defects, with no significant change in grain size. The stable layered structure indicates that the Cr/AlCrNbSiTiN/AlCrNbSiTiO multilayer coating has relatively good thermal stability, which ensures the coating’s optical performance. From the cross-sectional images of the annealed samples, it can be seen that the interface between the metallic Cr layer and the HEAN coating has become blurred, suggesting that heat treatment exerts a minimal impact on the microstructure of the Cr/AlCrNbSiTiN/AlCrNbSiTiO multilayer coating. The inter-diffusion at the coating/alloy interface should have a higher impact, considering the low thickness of the coatings [28]. At the same time, the sample with metallic coating had a lot of cracks at the coating/alloy interface which further accelerate the internal diffusion of oxygen and eventually lose their protective effect [29]. As depicted in the EDS mapping images of Si-ACNSTN-4 in Figure 4, the elements display a distinct layered distribution, which corroborates the multilayer architecture of the absorber coating. Significantly, it is observed that nitrogen is less prevalent in the outermost layer, and this phenomenon is ascribed to the existence of the oxide antireflection layer. Moreover, EDS spectroscopy validates the formation of the high-entropy phase. A more comprehensive microstructural analysis, encompassing high-resolution TEM, will be presented later in the manuscript.

**Figure 5:**
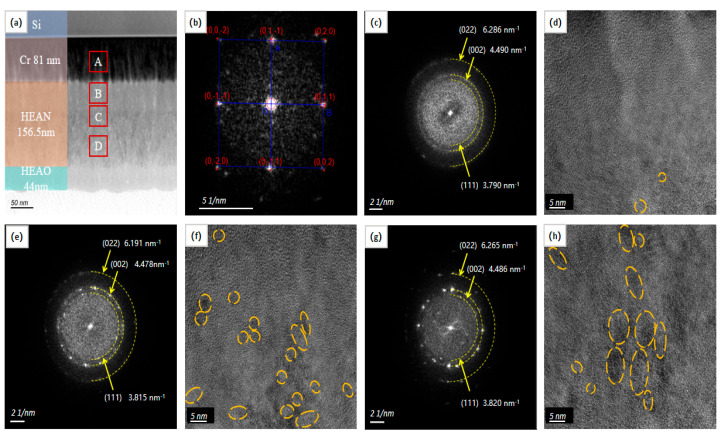
TEM images of the Si-ACNSTN-4 coating after 1 h of heat treatment at 500 °C under atmospheric conditions. (**a**) Cross-sectional bright-field image of the coating; (**b**) SAED pattern of region A in (**a**); (**c**,**d**) are the SAED and HRTEM images of region B in (**a**), respectively; (**e**,**f**) are the SAED and HRTEM images of region C in (**a**), respectively; (**g**,**h**) are the SAED and HRTEM images of region D in (**a**), respectively. The nanocrystalline phases exhibiting diffraction fringes are indicated by the orange dashed circles in (**d**,**f**,**h**) , while the remaining regions consist of the amorphous phase.

To further uncover the structural essence underpinning the coating’s ability to retain excellent performance in high-temperature environments, a systematic TEM analysis was carried out. Figure 5 displays the cross-sectional TEM results of the Si-ACNSTN-4 coating following 1 h of heat treatment at 500 °C under atmospheric conditions. Figure 5a presents the bright-field image of the coating, which features a clearly defined layered structure: the bottom Cr infrared reflective layer, the middle AlCrNbSiTiN absorption layer, and the topmost AlCrNbSiTiO anti-reflective layer. Measurements show that the total thickness of the multilayer coating sample is controlled within the optimized range of 282 nm, with the Cr layer measuring 81 nm, the AlCrNbSiTiN layer 156.5 nm, and the AlCrNbSiTiO layer 44 nm. Notably, the Cr metallic reflective layer is composed of columnar crystal structures grown with a preferred orientation along the <011> direction (Figure 5b). The AlCrNbSiTiN absorption layer exhibits gradient nanostructural characteristics: the region close to the substrate is fully amorphous, while the region farther from the substrate forms a distinctive nanocrystalline-amorphous nanocomposite structure. This structure consists of high-entropy nitride nanocrystals-ranging in size from 1 to 13 nm (highlighted by yellow circles in Figure 5d,f,h—embedded within an amorphous matrix).

Under the selected area electron diffraction (SAED) mode, it can be observed that in regions B, C, and D of Figure 5c,e,g, despite slight variations in the intensity of diffraction spots within the diffraction rings, all spots correspond to the (111), (002), and (022) crystal planes of the face-centered cubic (fcc) structure. This confirms that these three regions share the same high-entropy phase composition. The presence of diffraction rings indicates a large amount of crystalline phase in the coating. Additionally, the SAED patterns feature a central bright spot and diffraction rings that weaken outward, further confirming the presence of an amorphous phase in the coating. These observations suggest that the multi-layer coating has a structure combining nanocrystalline and amorphous phases. In high-resolution HRTEM images, the coexistence of lattice fringes and disordered regions is observed, confirming the “nanocrystalline-amorphous” dual-phase structure. Furthermore, a comparison of Figure 5c,e,g reveals that as one approaches the surface of the coating, the intensity of diffraction rings decreases while more independent diffraction spots appear. This indicates that the microstructure transitions from the substrate toward the surface: the nanocrystalline phase content increases, grain size gradually coarsens, and the amorphous phase content decreases. This aligns with the design concept of gradient coatings.

### 3.3. Phase Composition of the Coatings

**Figure 6:**
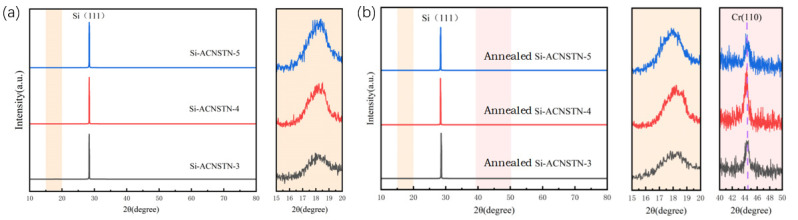
XRD patterns of the as-deposited (**a**) and 500 °C 1 h heat-treated (**b**) Cr/AlCrNbSiTiN/AlCrNbSiTiO multilayer coatings.

The XRD spectra in Figure 6 show the Cr/AlCrNbSiTiN/AlCrNbSiTiO multilayer coatings on Si substrates before and after heat treatment. In Figure 6a, it is clear that in the as-deposited state, all three samples display distinct characteristic peaks at 2θ = 28.4^o^ corresponding to the (111) crystal plane of silicon. Comparison with the standard PDF#01-070-5680 confirms the peak positions, indicating that this signal likely originates from the silicon substrate. By magnifying the 2θ = 15°–20° section, it can be observed that all three samples have obvious low-intensity broad peaks, which might be caused by nitride nanocrystals (grain size < 10 nm) [30]. By comparing the X-ray diffraction patterns of the three samples, it can be seen that as the deposition time of AlCrNbSiTiN under a magnetron power of 100 W increases, the intensity of the broadened peaks gradually increases, which may be due to the increase in the number of nanocrystals generated within the multilayer coating. Figure 6b shows the XRD spectra of the samples after heat treatment. All three samples exhibit prominent diffraction peaks corresponding to the Si (111) crystal plane at 2θ = 28.4°. A closer inspection of the 2θ range from 15° to 20° reveals that the low-intensity, broadened peak persists, though its intensity is slightly reduced compared to the pre-annealing state. Further magnification of the 2θ range between 40° and 50° uncovers distinct characteristic peaks near 2θ = 44° for Si-ACNSTN-3, Si-ACNSTN-4, and Si-ACNSTN-5. By comparing these peaks with the PDF#06-0694 card, they are confirmed to be characteristic of the Cr (100) crystal plane. The emergence of new characteristic peaks after annealing can be attributed to segregation crystallization of Cr atoms in the amorphous regions, which overcome energy barriers under thermal energy, forming long-range ordered crystalline structures and completing the phase transition from the amorphous to the crystalline state [16].

### 3.4. Chemical States of the Coatings

**Figure 7:**
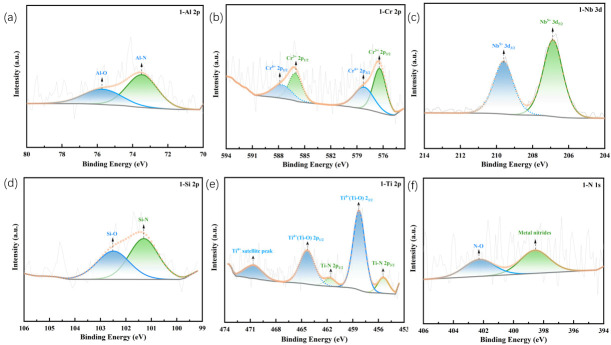
XPS spectra of the Cr-ACNSTN-4 multilayer coating: (**a**) Al 2p, (**b**) Cr 2p, (**c**) Nb 3d, (**d**) Si 2p, (**e**) Ti 2p, (**f**) N 1s.

The XPS spectra of the as-deposited sample are presented in Figure 7, showing the coexistence of elements Al, Cr, Nb, Si, Ti, N, O, and C with no other impurities detected. Peak fitting analysis was conducted for each element. The Al 2p spectrum in Figure 7a reveals that aluminum exists in the material in two forms: 36.86% Al-O (73.46 eV) and 63.14% Al-N (75.69 eV). The Al-N component enhances high-temperature stability, while Al-O forms a passivation film to improve thermal stability. In the as-deposited material, Cr (as shown in Figure 7b) mainly exists in a mixed valence state of Cr^3+^ (576.41 eV, 586.07 eV) and Cr^6+^ (578.19 eV, 587.6 eV) with an approximate ratio of 1:1. This mixed valence structure may enhance the visible light absorption performance through multivalent state transitions [22,24]. Nb (as seen in Figure 7c) exists entirely in the Nb^5+^ valence state (206.87 eV, 209.61 eV), providing chemical inertness protection. Silicon (shown in Figure 7d) primarily exists in the form of Si-O bonds (102.49 eV) and Si-N bonds (101.3 eV). In the Ti 2p spectrum (Figure 7e), 84.55% of titanium is present in the Ti^4+^ chemical state (Ti-O) corresponding to binding energies of 458.27 eV and 464.33 eV, indicating oxidation occurred during the preparation process. The N 1s fine spectrum (Figure 7f) shows the presence of Metal-N (398.58 eV) and N-O (402.28 eV) bonding forms, with relative atomic contents of 57.16% and 42.84% respectively, confirming the coexistence of nitride and oxide components in the material.

**Figure 8:**
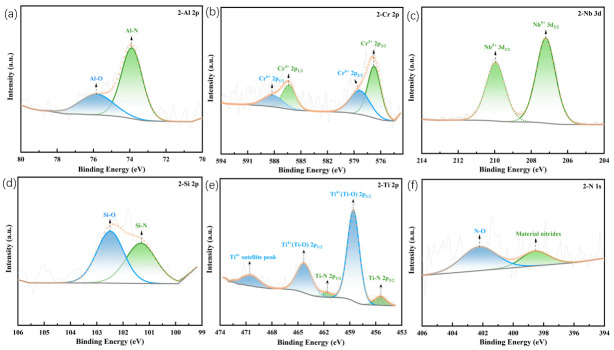
XPS patterns of the Cr-ACNSTN-4 multilayer coating after annealing: (**a**) Al 2p, (**b**) Cr 2p, (**c**) Nb 3d, (**d**) Si 2p, (**e**) Ti 2p, (**f**) N 1s.

Figure 8 presents the XPS test results of the annealed Cr-ACNSTN-4 coating. After annealing in air at 500 °C for 1 h, notable changes were observed: As shown in Figure 8a, the proportion of Al-N bonds increased to 67.83% with a reduced full width at half maximum, suggesting enhanced crystallinity that helps suppress high-temperature diffusion. In Figure 8b, Cr retained its mixed valence state, though the proportion of Cr^6+^ rose slightly to 53.36–the d-d transition of Cr^6+^ is expected to broaden the visible light absorption range. Figure 8c reveals a minor positive shift (approximately 0.3 eV) in the binding energy of Nb^5+^. As depicted in Figure 8d, the content of Si-O bonds exceeded that of Si-N bonds, reaching 53.47%; while the SiO_2_ network improves thermal shock resistance, it may compromise interfacial bonding. The oxidation of Ti (Figure 8e) intensified further, with the proportion of Ti-O bonds increasing from 84.55% to 91.66% and Ti-N bonds decreasing accordingly. In the N 1s spectrum (Figure 8f), the content of Metal-N bonds dropped sharply to 32.98%, whereas N-O bonds surged to 67.02%, indicating that annealing significantly facilitated the phase transition from nitrides to oxides.

## 4. Conclusions

This work fabricates multilayer gradient Cr/AlCrNbSiTiN/AlCrNbSiTiO coatings via magnetron sputtering and investigates their chemical states, optical properties, and photothermal conversion performance both before and after annealing. The Si-ACNSTN-3, Si-ACNSTN-4, and Si-ACNSTN-5 coatings exhibit absorption rates of 98.47%, 98.88%, and 98.79% respectively, demonstrating excellent spectral absorption performance. After heat treatment at 500 °C for 1 h in an air atmosphere, the spectral absorption rate of the Si-ACNSTN-3 sample increased by 0.21%, while those of Si-ACNSTN-4 and Si-ACNSTN-5 decreased by 0.24% and 0.29% respectively. Under standard solar radiation, the maximum surface temperatures of the three samples reached 58.9 °C, 61.6 °C, and 65.1 °C. Post-annealing, the intensity of the broad amorphous peak diminished, and diffraction peaks corresponding to the Cr (110) plane emerged, indicating that the Cr atoms in the coating samples underwent a phase transition from amorphous to crystalline due to the elevated temperature. TEM analysis revealed that the coating samples possess a distinct nanocrystalline-amorphous structure, with the absorption layer conforming to the classic face-centered cubic nitride structure. The presence of metal nitrides endows the multilayer coatings with favorable light absorption characteristics, while the oxide phases ensure their thermal stability and environmental durability. The mixed valence states of Cr^3+^/Cr^6+^ can enhance absorption efficiency in the visible light region through a multi-electron transition mechanism.

## Figures and Tables

**Table 1:** Deposition parameters.

Coatings	Working Pressures /Pa	Temperature /°C	Bias Voltage /V	Ion Source Power /W	Magnetron Powers /W	Deposition Time/min
Cr	0.89	100	−100	35	120	2
AlCrNbSiTiN	1.16	100	−100	35	100	3/4/5
AlCrNbSiTiN	1.16	100	−100	35	80	2
AlCrNbSiTiO	1.18	100	−100	35	80	2

## Data Availability

The original contributions presented in this study are included in the article. Further inquiries can be directed to the corresponding authors.
